# Supplementary material for: Spin Qubits Candidate in Transition-Metal-Ion doped Halide Double Perovskites
Source: Nat Commun. 2026 Jan 8;17:415. doi: 10.1038/s41467-025-67980-2 (PMC12796413; doi:10.1038/s41467-025-67980-2)
Supplement: Supplementary file 1 — Supplementary Information [file 41467_2025_67980_MOESM1_ESM.pdf]

Supporting Information for

Spin Qubits Candidate in Transition-Metal-Ion doped Halide Double Perovskites

Sakarn Khamkaeo[1], Kunpot Mopoung[1], Kingshuk Mukhuti[2], Maarten W. de Dreu[2], Anna Dávid[3], Muyi Zhang[1], Mats Fahlman[3], Feng Gao[1], Peter C.M. Christianen[2], Irina A. Buyanova[1], Weimin M. Chen[1]* and Yuttapoom Puttisong[1]*

[1]Department of Physics, Chemistry and Biology (IFM), Linköping University, Linköping, Sweden

[2]HFML-FELIX, Toernooiveld 7, 6525ED Nijmegen, the Netherlands; Institute for Molecules and Materials, Radboud University, Heyendaalseweg 135, 6525 AJ Nijmegen, the Netherlands

[3]Laboratory of Organic Electronics (LOE), Department of Science and Technology, Linköping University, 60174 Norrköping, Sweden.

Corresponding Author's e-mail: weimin.chen@liu.se, yuttapoom.puttisong@liu.se

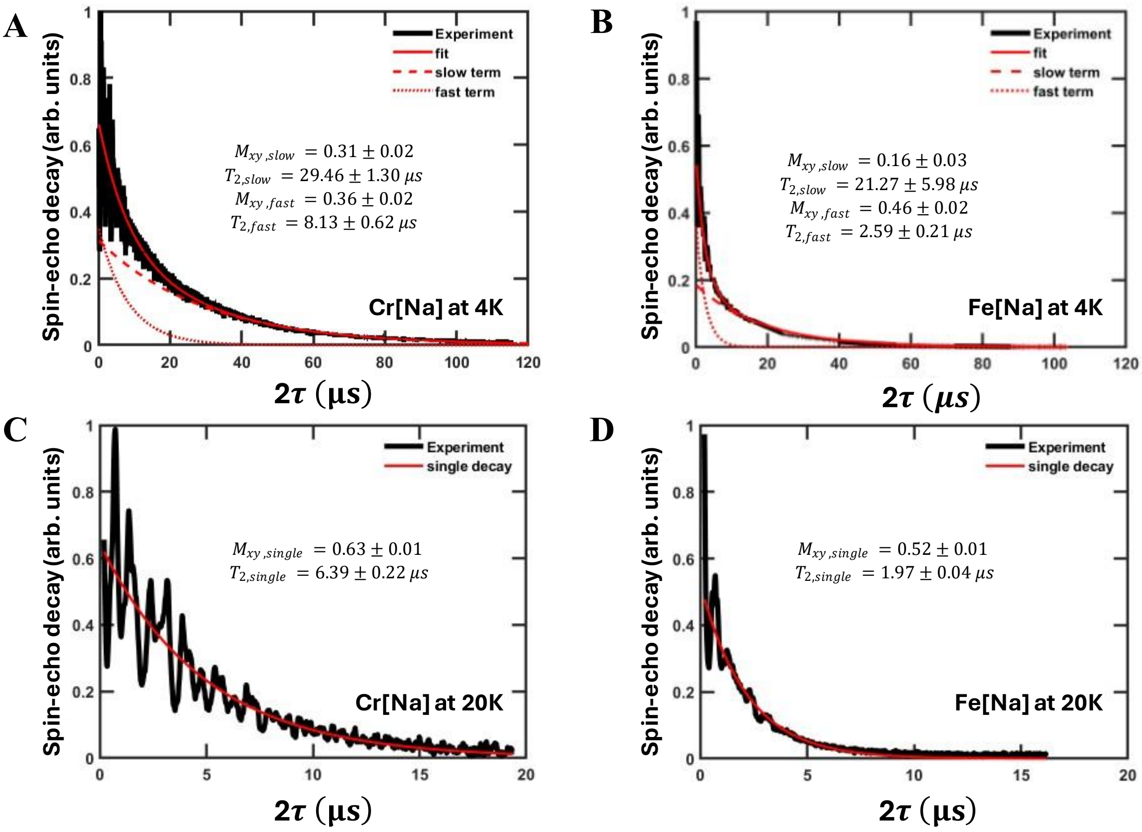

Figure S1: Exponential fitting of spin-echo signals. **A** Cr[Na] – Cr^{3+} in $Cs_2NaInCl_6$ at 4K **B** Fe[Na] – Fe^{3+} in $Cs_2AgInCl_6$ at 4K **C** Cr[Na] at 20K and **D** Fe[Na] at 20K. The fitting was performed with the function $I_{echo} = M_{xy}e^{2\tau/T_2}$, where M_{xy} is the in-plane magnetization. The fitting employs two exponential terms in A and B and the single exponential term in C and D.

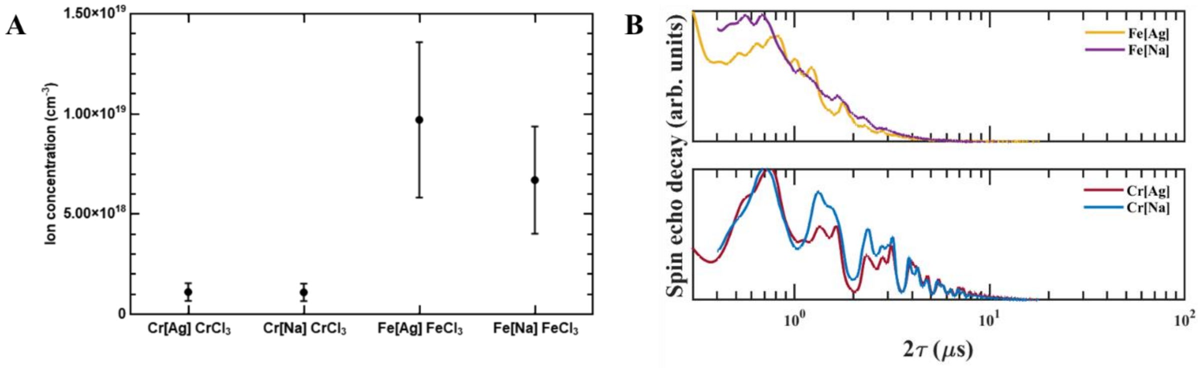

Figure S2. The actual concentration of TM in HDPs host and their spin echo decay. A Actual TM ion (Fe^{3+} and Cr^{3+}) concentrations in both $Cs_2NaInCl_6$ and $Cs_2AgInCl_6$ hosts. **B** (top) Spin echo decay of Fe[Ag] and Fe[Na] (top), and Cr[Ag] and Cr[Na] (bottom) with the ion concentrations given in A.

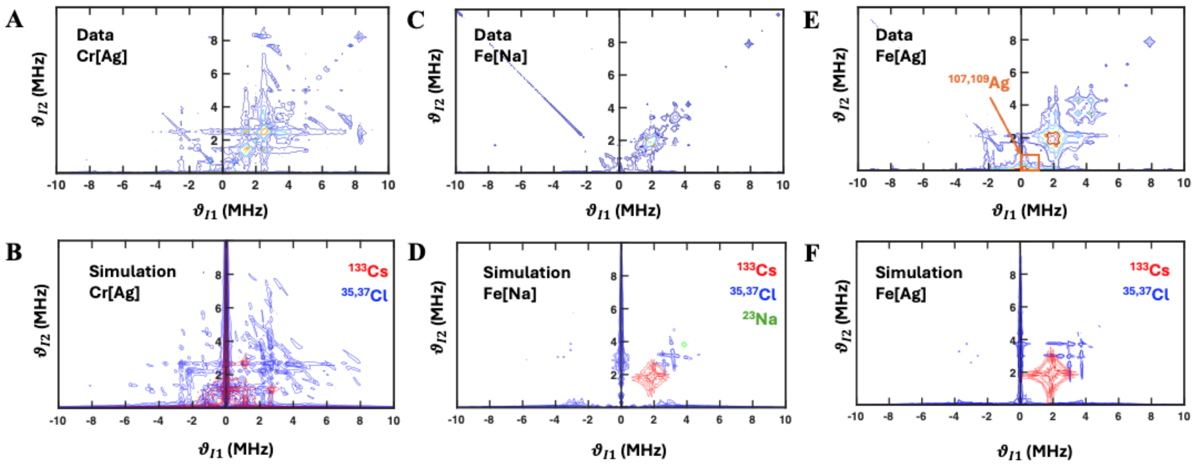

Figure S3 Frequency domain HYSCORE spectra and HYSCORE simulations A, B Cr^{3+} centers in $Cs_2AgInCl_6$. **C, D** Fe^{3+} centers in $Cs_2NaInCl_6$, and **E, F** Fe^{3+} centers in $Cs_2AgInCl_6$, performed at 4K. The fitting parameters are given in the Table S1.

Table S1. Fitting parameters from HYSCORE spectra

1.1 Cr^{3+} spin qubits in $Cs_2NaInCl_6$

Nuclear	Spin (I)	Hyperfine tensors (A_x,A_y,A_z) MHz	Quadrupole tensors (Q_x,Q_y,Q_z) MHz	Principle axis
^{133}Cs	7/2	(0.94, 0.94, 1.42) ±0.3	0	[111]
^{35}Cl	3/2	(0.42, 0.42, -7.12) ±0.3	(-2.01, -2.01, 4.02) ±0.3	[001]
^{37}Cl	3/2	(0.35 0.32 -5.93) ±0.3	(-1.68 -1.68 3.35) ±0.3	[001]
Na	3/2	NaN	NaN	NaN

1.2 Cr^{3+} spin qubits in $Cs_2AgInCl_6$

Nuclear	Spin (I)	Hyperfine tensors (A_x,A_y,A_z) MHz	Quadrupole tensors (Q_x,Q_y,Q_z) MHz	Principle axis
^{133}Cs	7/2	(1.42, 1.42, 1.8) ±0.3	0	[111]
^{35}Cl	3/2	(0.2, 0.2, -7.4) ±0.3	(-0.60, -0.60, 1.20) ±0.3	[001]
^{37}Cl	3/2	(0.17, 0.17, -6.17) ±0.3	(-0.50, -0.50, 1.00) ±0.3	[001]
$^{107,109}Ag$	½	NaN	NaN	[001]

1.3 Fe^{3+} spin qubits in $Cs_2NaInCl_6$

Nuclear	Spin (I)	Hyperfine tensors (A_x,A_y,A_z) MHz	Quadrupole tensors (Q_x,Q_y,Q_z) MHz	Principle axis
^{133}Cs	7/2	(0.1, 0.1, 0.2)*	0	[111]
^{35}Cl	3/2	(0.6, 0.6, -7.8) ±0.2	(-1.33, -1.33, 2.66) ±0.2	[001]
^{37}Cl	3/2	(0.5, 0.5, -6.5) ±0.2	(-1.11, -1.11, 2.22) ±0.2	[001]
Na	3/2	weak	weak	[001]

1.4 Fe^{3+} spin qubits in $Cs_2AgInCl_6$

Nuclear	Spin (I)	Hyperfine tensors (A_x,A_y,A_z) MHz	Quadrupole tensors (Q_x,Q_y,Q_z) MHz	Principle axis
^{133}Cs	7/2	(0.1, 0.1, 0.2)*	0	[111]
^{35}Cl	3/2	(1.2 1.2 -9.5) ±0.2	(-1, -1, 2) ±0.2	[001]
^{37}Cl	3/2	(1 1 -7.92) ±0.2	(-0.83, -0.83, 1.66) ±0.2	[001]
Ag	1/2	NaN	NaN	[001]

*the upper bound limit

Supplemental Note 1: Modeling electron spin localization

The Fermi contact interaction is the part of hyperfine interaction of electrons in approximately spherical-like orbital and surrounded nuclear environment. It can be expressed by:

$$A_{iso} = \frac{2}{3}\mu_0 g_e \beta_e g_N \beta_N |\Psi|^2$$

This can also demonstrate the electron spin localization, where the percentage of electron density, $|\Psi|^2$, distributing at the neighbor atoms is estimated by calculating the ratio between electron density distribution caused by hyperfine interaction and free electron localized at each nuclear site:

$$\frac{|\Psi_{hyperfine}|^2}{|\Psi_{free}|^2} = \frac{|A_{iso,experiment}|}{|A_{iso,free}|}$$

In our case, we consider 3 nuclear sites representing the nearest neighbours (^{35}Cl and ^{37}Cl) and the next-nearest neighbours (^{133}Cs) of incorporated transition metal. By inserting hyperfine coupling strength, we can obtain the percentage of electron localization at each nuclear site (See Figure R1 and Table R1).

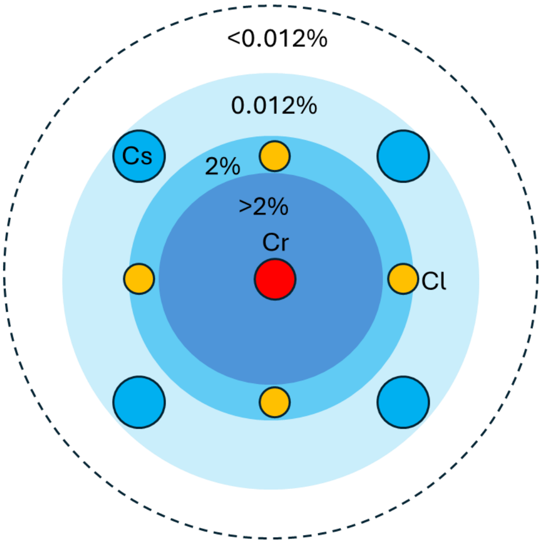

Figure S4: The example illustration of electron density localization at each nuclear site (^{35,37}Cl, ^{133}Cs) around the incorporated Cr^{3+}.

Table S2: Percentage of electron localization from Fermi contact interaction

Nuclear site	$A_{iso,experiment} = \frac{A_x + A_y + A_z}{3}$ (MHz)	$A_{iso,free}$ (MHz)	% electron localization
Cr[Na]			
^{133}Cs	1.1	9.2x10^3	0.012%
^{35}Cl	-2.1	80	2.6%
^{37}Cl	-1.6	66	2.5%
Na	-	-	-

Cr[Ag]			
^{133}Cs	1.5	$9.2x10^3$	0.017%
^{35}Cl	-2.3	80	2.9%
^{37}Cl	-1.8	66	2.7%
Ag	-	-	-
Fe[Na]			
^{133}Cs	0.1	$9.2x10^3$	0.001%
^{35}Cl	-2.2	80	2.8%
^{37}Cl	-1.7	66	2.6%
Na	-	-	-
Fe[Ag]			
^{133}Cs	0.1	$9.2x10^3$	0.001%
^{35}Cl	-2.4	80	3.0%
^{37}Cl	-1.8	66	2.8%
Ag	-	-	-

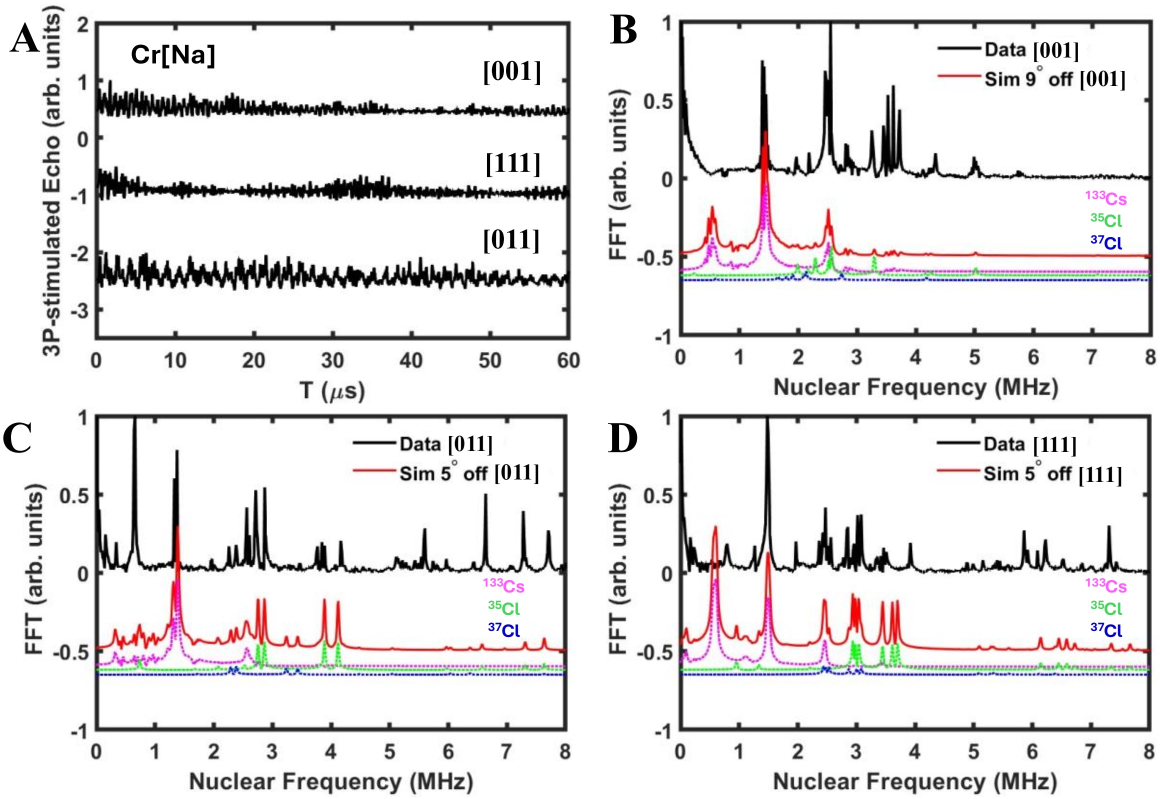

Figure S5. Results and simulation of stimulated echo spectra. A Stimulated echo decay with the magnetic field (B) aligned along the [001], [111], and [011] crystallographic directions. **B-D** Nuclear frequency spectra obtained from 3-pulse stimulated echo, with B aligned along the [001], [011], and [111] directions. Simulations of the orientation-dependent nuclear frequencies of ^{133}Cs and ^{35,37}Cl are shown assuming a slight misalignment to account for experimental error that leads to the peak splitting arising from magnetically inequivalent sites within the O_h point group symmetry.

Supplemental Note2: Schematic energy level diagrams for the nuclear spin rotation frequency of the Cr^{3+} spin centers coupled with the ^{133}Cs nuclear spin (I =7/2)

The energy diagram shown in **Fig. S6** illustrates how nuclear spin rotation frequencies are determined for the Cr^{3+} spin qubits (S = 3/2) coupled to the ^{133}Cs nuclear spins (I = 7/2).

<u>Hyperfine and Zeeman Interactions:</u> Each electron spin sublevel (m_s) interacts with nuclear spin sublevels (m_I) through the hyperfine interaction. The nuclear frequency splitting is influenced by both this hyperfine interaction and the nuclear Zeeman interaction. Consequently, the largest splitting occurs between adjacent nuclear spin states within the m_s = -3/2 sublevel, while the smallest occurs within the m_s = +3/2 sublevel.

<u>Single Quantum Transitions:</u> Allowed nuclear spin transitions occur between adjacent energy levels (Δm_I=±1) and are probed via allowed electron spin resonance transitions (Δm_s=±1). These transitions, known as single quantum (SQ) transitions, are indicated by the light blue areas in the diagram.

<u>The Dominant Nuclear Spin Rotation Frequencies</u>**:** Because ^{133}Cs lacks quadrupole splitting, each nuclear spin rotation within the I = 7/2 manifold has a similar frequency. Therefore, the interaction between Cr^{3+} (S = 3/2) and ^{133}Cs (I = 7/2) results in four dominant nuclear rotation frequencies. These are separated by the hyperfine parameter (A) along the direction of the magnetic field (B) and are centered around the fundamental Larmor frequency of ^{133}Cs (1.9458 MHz).The experimental data also reveals nuclear double quantum (NDQ) rotations (ΔM_s = ±1, Δm_I = ±2), as highlighted by the red arrow in Fig. S6.

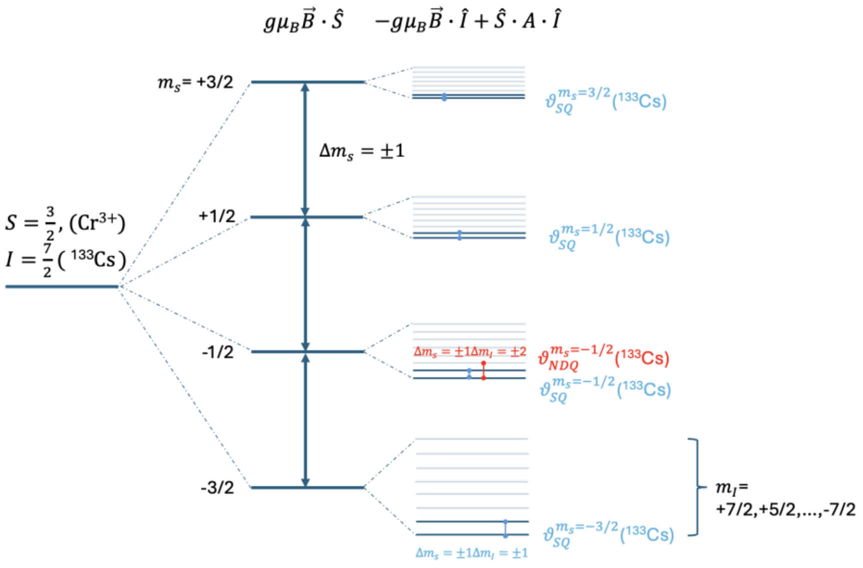

Figure S6. Energy level diagram for a system with electron spin S = 3/2 and nuclear spin I = 7/2 in a magnetic field with hyperfine coupling. The light blue arrow indicates a single quantum transition, while the red arrow marks a nuclear double quantum transition.

Supplemental Note 3: Target addressing ^{133}Cs nuclear spin in the single crystalline samples

This approach is based on controlling the degree of electron-nuclear spin mixing, which causes the undulation of the pulsed-EPR signals. The degree of this mixing is highly dependent on the orientation of the external magnetic field (B) relative to the principal axes of the hyperfine tensor. The modulation depth of the electron spin coherence, which is a measure of the degree of nuclear spin mixing, can be estimated using the following formula:

$$k = \frac{A_{nonsecular}^2}{\omega_I^2 + A_{secular}^2}$$

Where, ω_I is the nuclear spin frequency. The secular and nonsecular terms of the hyperfine tensor (in the LAB frame) are extracted from the hyperfine tensor in principal axis form $\boldsymbol{A_{principal}} = (A_x, A_y, A_z)$. In our case, let the external field point to the z-axis, so that $A_x = A_y = A_\perp$ and $A_z = A_\parallel$.

By rotating the $\boldsymbol{A_{principal}}$ to the LAB frame using the Euler angle rotation matrix, we will get

$$A_{secular} = A_\perp \sin^2\theta + A_\parallel \cos^2\theta$$
$$A_{nonsecular} = (A_\parallel - A_\perp) sin\theta cos\theta$$

where θ is the angle between the external magnetic field vector and the principal z-axis of the hyperfine tensor. When the magnetic field is aligned along the principal axis (e.g., the <001> direction), the non-secular term approaches zero, effectively "turning off" the spin mixing for that specific nuclear species.

We leverage this effect to demonstrate targeted nuclear spin addressing. For example, by aligning the magnetic field along the <001> direction, the ^{35,37}Cl nuclear spins' contribution to spin mixing is suppressed ($A_{nonsecular}$ = 0), allowing the ^{133}Cs spins to be addressed as the sole target of nuclear spin registration. This interaction is deterministic because the number of available sites is reduced to the four magnetically equivalent sites of the eight Cs atoms. Furthermore, slightly rotating the magnetic field off the <001> direction allows us to distinguish between these four different magnetically equivalent Cs sites (Cs1, Cs2, Cs3, Cs4, as shown in the inset of Figure 4 in the main text), which is the key for addressing individual nuclear spins (or a pair of nuclear spin in our case).

Supplemental Note 4: Identify Optical Signature of the Cr^{3+} spin centers

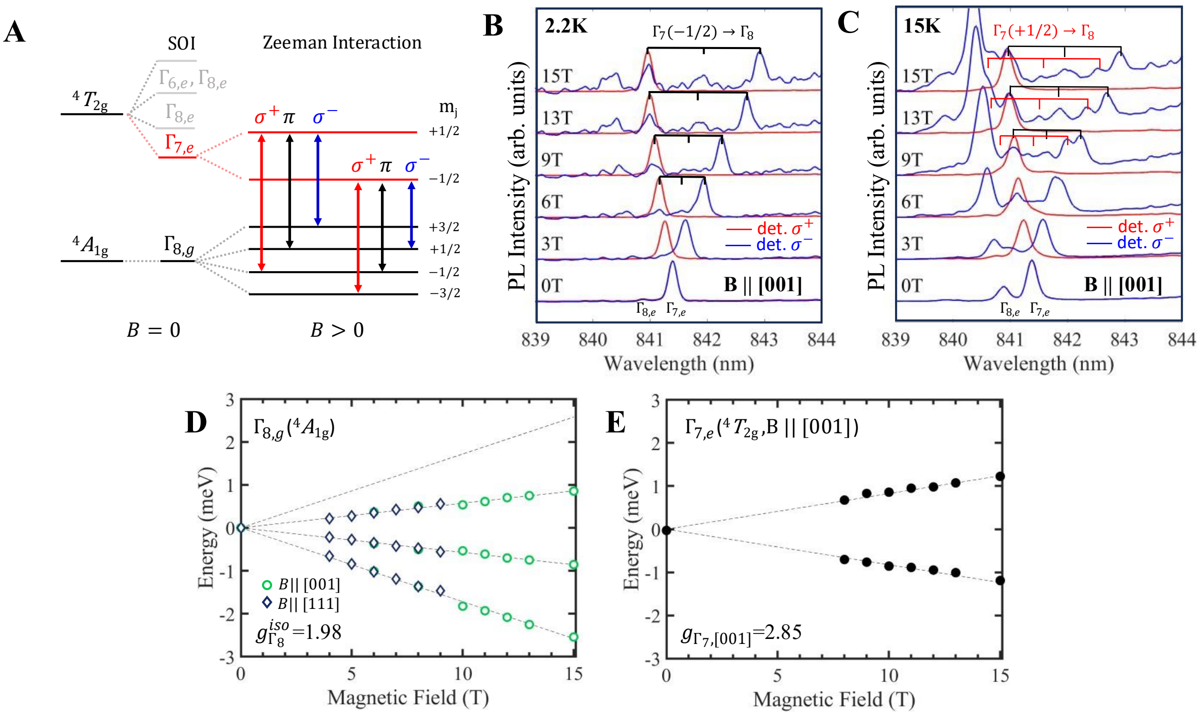

Figure S7 Optical transitions and magneto-PL spectrum of Cr^{3+} doped $Cs_2NaInCl_6$. **A** The energy diagram of electronic states of the Cr^{3+} ion in the cubic crystal field. The energy levels are split further by spin-orbit interaction (SOI) and the Zeeman effect. The arrows indicate optical transitions between the grown state and the lower/upper (black/red arrows) spin sublevels of the first excited state. Each transition consists of three sub-transitions following the selection rules: $\Delta m_s = \pm 1$ for $\sigma^{\pm}$ and $\Delta m_s = 0$ for π. **B-C** The magneto-PL spectra obtained at 2.2K and 15K. The red and blue solid lines correspond to σ^+ and σ^- detections, respectively. The black brackets indicate the peaks related to the transition from the $|-\frac{1}{2}>$ excited state to the ground state, which dominates at 2.2 K and reflect the splitting of the Γ_8 ground state. The red brackets indicate the peaks related to the transition from the $|+\frac{1}{2}>$ excited state to the ground state. The energy separation between these two sets thus refelects the splitting of the Γ_7 excited state. **D** Peak marking from magneto-PL results plotted with the energy splitting of the Γ_8 ground state, calculated from $E = m_j g \mu_B B$, where $m_j = \pm\frac{1}{2}, \pm\frac{3}{2}$. The green circles are extracted from measurements with the magnetic field parallel to the [001] direction, while the blue diamonds are from measurements with the magnetic field parallel to the [111] direction. This plot demonstrates that the g-factor of ground state is isotropic. **E** Peak marking results plotted with the energy splitting of the Γ_7 excited state. This has a parallel component of g-factor, $g_{\Gamma_{7,<001>}} = 2.85$ and $m_j = \pm\frac{1}{2}$. The black dots follow the calculated Zeeman splitting, confirming the splitting of the excited states shown in the energy diagram.

The electronic structures governing the $^4T_{2g} \rightarrow {}^4A_{1g}$ transition of the Cr^{3+} ions are illustrated in **Fig. S7A**. Under the spin-orbit interaction (SOI) to the second order, the orbitally non-degenerate $^4A_{1g}$ ground state retains its four-fold spin character, corresponding to a Γ_8

representation. The $^4T_{2g}$ excited state, on the other hand, splits into four sublevels. The lowest-lying excited states, a Γ_7 doublet, is instrumental for the spin-selective optical protocols.

Applying an external magnetic field (B > 0) lifts the remaining degeneracies via the Zeeman effect, splitting the $^4A_{1g}$ ground state into four sublevels (m_j = ±1/2, ±3/2) and the Γ_7 excited state into two sublevels (m_j = ±1/2). The optical transitions between these sublevels are governed by selection rules. Transitions with $\Delta m_j = 0$ correspond to light polarized linearly along the magnetic field (π), while $\Delta m_j = \pm 1$ transitions correspond to circularly polarized light (σ±). Transitions with $\Delta m_j = \pm 2$ are optically forbidden.

To validate the proposed electronic structure and polarization selectivity, we performed polarization-resolved magneto-photoluminescence (PL) experiments on the zero-phonon lines (ZPLs), as shown in Figs. S9B (2.2 K) and S9C (15 K). At 2.2 K, the PL is dominated by the $\Gamma_{7,e} \rightarrow \Gamma_{8,g}$ transition at 841.1 nm. Under an applied magnetic field (B > 3 T), emission originates primarily from the thermally populated lowest excited sublevel, $\Gamma_{7,e}$(-1/2) and splits into three resolvable Zeeman lines, marked in black in Fig. S9B. The number of observed transitions, their energy positions, and their distinct polarizations provide strong evidence for our model (Fig. S9A). Specifically, the lowest-energy peak corresponds to the $\Gamma_{7,e}$(-1/2) $\rightarrow \Gamma_{8,g}$ (+1/2) which is σ^- polarized, while the $\Gamma_{7,e}$(-1/2) $\rightarrow \Gamma_{8,g}$ (-3/2) is σ^+ polarized. The intermediate line is weak, as expected for a transition whose electric-field vector is parallel to the detection axis.

Upon warming to 15 K, thermal energy activates the transitions from the higher-lying states, including the next excited state $\Gamma_{8,e}$ (at 839.9 nm), and importantly, the $\Gamma_{7,e}$(+1/2) sublevel marked in red in Fig S7C. The appearance of these additional transitions in the magneto-PL spectrum allows us to unambiguously identify the energy levels, providing further corroboration for our assignments.

The field-dependent data at 2.2 K, where the emission originates solely from the Γ_7(-1/2) sublevel, allow for a precise determination of the $^4A_{1g}$ ground state g-factor from the Zeeman data. Fitting the Zeeman splitting for magnetic fields along both the [001] and [111] crystallographic directions yield an isotropic g_{iso} = 1.98, which is shown in Fig. S7D. This value is identical to that determined from independent spin resonance experiments, providing strong evidence that the optical transition is directly coupled to our spin qubit probed in the pulsed EPR experiments. With the ground-state g-factor established, an analysis of the magneto-PL data yields an excited state g-factor of g(Γ_7) = 2.86 through the fitting in Fig. S7E.

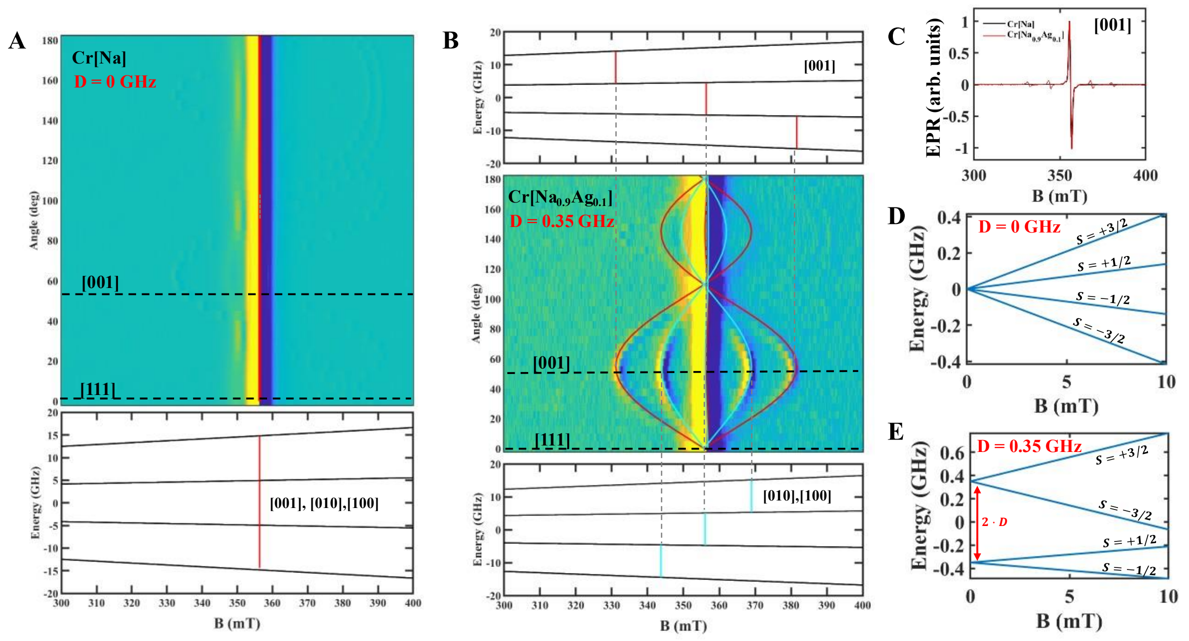

Figure S8 EPR spectra of Cr3+ in B^I-site alloy hosts. A-B. Room temperature, angular-dependent Cr^{3+} EPR spectra of $Cs_2NaInCl_6$ single crystal and $Cs_2Na_{0.9}Ag_{0.1}InCl_6$ single crystal, when the magnetic field was rotated in the (1-10) plane. The energies of the spin sublevels with the magnetic field pointing along the [001], [010] and [100] axes are also shown. The red and cyan is the simulation fitting with different magnetically inequivalent sites of the tetragonal symmetry. **C.** The EPR spectra along a [001] direction taken from both samples a room temperature. **D** and **E**, the energy diagram near zero magnetic field for the cubic and tetragonal Cr^{3+} centers, respectively.

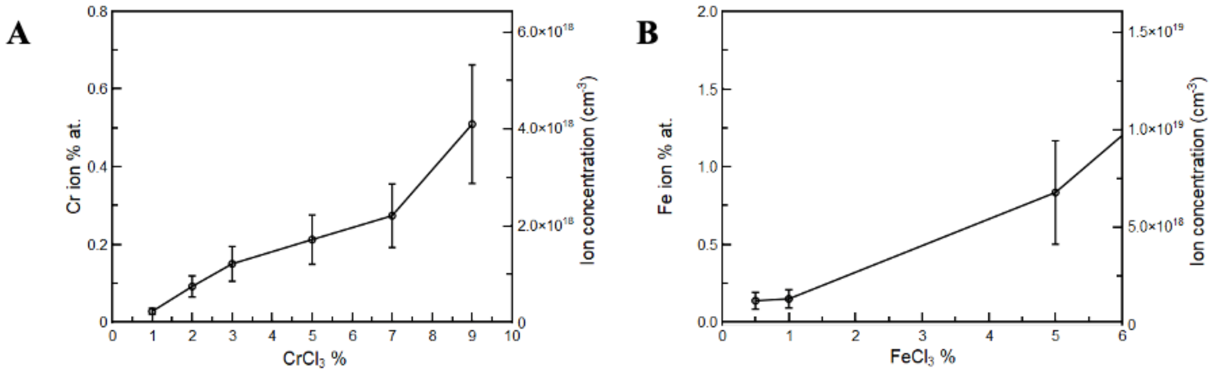

figure S9A-B Relation between the $CrCl_3$($FeCl_3$) precursor percentage and the actual Cr^{3+}(Fe^{3+}) incorporation in $Cs_2NaInCl_6$.

Supplemental Note 5: Pulsed electron spin resonance experiments

Our pulsed experiments were conducted under the hard-pulse condition, with π/2 and π pulse length equal to 16 ns and 32 ns (20ns and 40 ns in some cases to minimize pulse leak), respectively. Only in the Rabi nutation experiments, we fine-tune the pulse length to match π/2 and π condition at a given microwave power. The short repetition time (SRT) is set to match the condition SRT exceeds T_1 spin relaxation time. Our time step is limited to 4 ns at the minimum, which represents the bandwidth of 250 MHz.

Field-sweep Hahn echo: the measurements were performed with the (π/2 - τ - π - τ - echo) sequence. The first-time delay was set at 150-200 ns to avoid the excitation pulse. The echo was integrated at 2/3 of echo maxima to avoid the background noise. Up to 40 shots per point were collected to improve the signal-to-noise ratio. The echo signals were phase optimized. The B-field was scanned over the resonant field of the TM spin qubits over the range of 330-360 mT. The basic principle of Hahn-echo is shown in **Figure S10**.

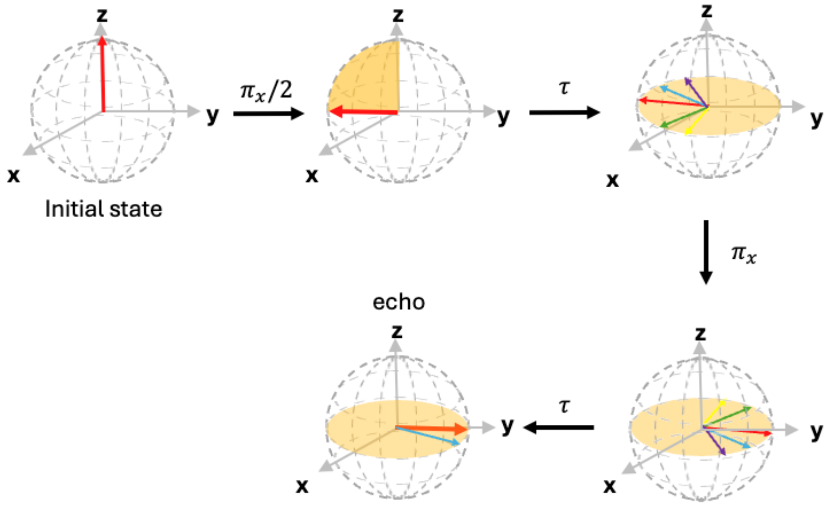

Figure S10. The evolution of electron spin coherence during a Hahn-echo experiment, where spins initially aligned along the z-axis are rotated into the x-y plane by the first π/2 pulse. During the first delay τ, spins evolve freely, accumulating phase before the π pulse rotates them 180°. At time τ, the spin packet refocuses and the spin echo occurs. A net xy polarization (i.e. spin coherence) could not be completely refocused due to the instantaneous diffusion, spin diffusion and the intrinsic single electron decoherence, causing a drop of spin echo amplitude as a function of 2τ.

Three-pulse stimulated echo: the measurements were performed with the (π/2 - τ - π/2 - T1 - π/2 - τ – echo) sequence. The basis set-up follows the Hahn echo experiments. In addition, we perform four-step phase recycling to eliminate the contribution of the unwanted echo. The initial value of T1 is 300 ns. The basic principle of three-pulse stimulated echo is shown in **Figure S11**.

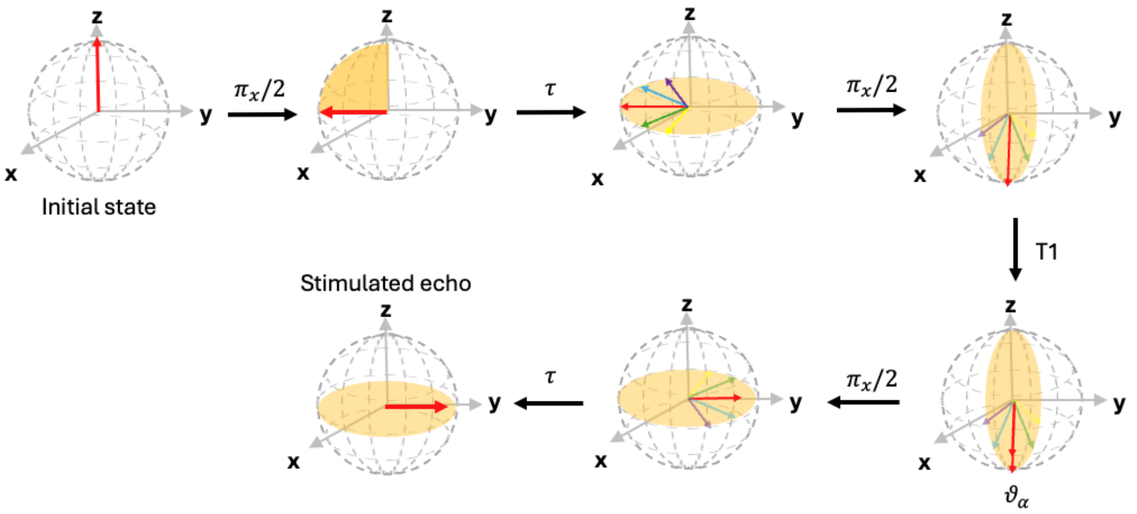

Figure S11. The evolution of electron spin coherence during a three-pulse stimulated echo experiment, where spins initially aligned along the z-axis are rotated into the x-y plane by the first π/2 pulse. During the first delay τ, spins evolve freely, accumulating phase before the second π/2 pulse rotates them into the x-z plane, converting coherence into polarization and encoding electron spin coherence into nuclear coherence via hyperfine interactions. During the subsequent delay T1, spins evolve under nuclear modulation, represented by spheres depicting spin packets with ϑ_α nuclear frequency rotation. The final π/2 pulse returns the spins to the x-y plane, restoring coherence and generating a stimulated echo at time τ.

Hyperfine Spin-sublevel Correlation (HYSCORE) is a two-dimension derivative of three pulse stimulated echo, with the sequence (π/2 - τ - π/2 - T1 - π – T2 - π/2 - τ – echo). The initial T1 and T2 is 300 ns. The basic principle of HYSCORE is described and shown in **Figure S12**.

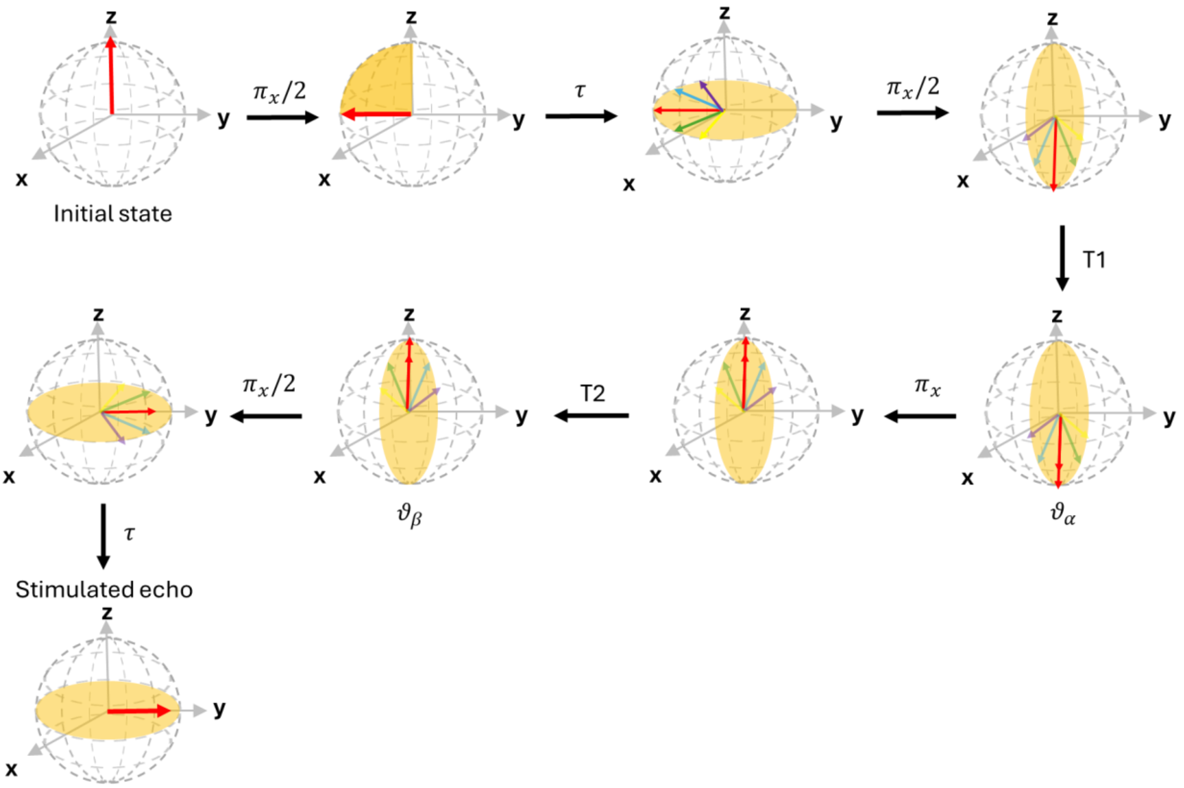

Figure S12. The evolution of electron spin coherence during the HYSCORE experiment, where spins initially aligned along the z-axis are rotated into the x-y plane by the first π/2 pulse. During the first delay τ, spins evolve freely, accumulating phase before the second π/2 pulse rotates them into the x-z plane, converting coherence into polarization and encoding electron spin coherence into nuclear coherence via hyperfine interactions. During the subsequent delay T1, spins evolve under nuclear modulation ϑ_α. A subsequent π pulse flips the spins around the x-axis. This transfers the polarization between two adjacent electron Zeeman sublevels, where the nuclear spin rotation is transformed to rotate with ϑ_β due to time evolution T2. The final π/2 pulse after time T2 returns the spins to the x-y plane, restoring coherence and generating a stimulated echo at time τ.

Echo detected nutation experiments were performed with the (x-nutation – T > T_2 - π/2 -τ - π - τ – echo) sequence. The basis setup follows the Hahn echo experiments, but the z polarization is prepared by the x-nutation pulse. The basic principle of three-pulse stimulated echo is shown in **Figure S13**.

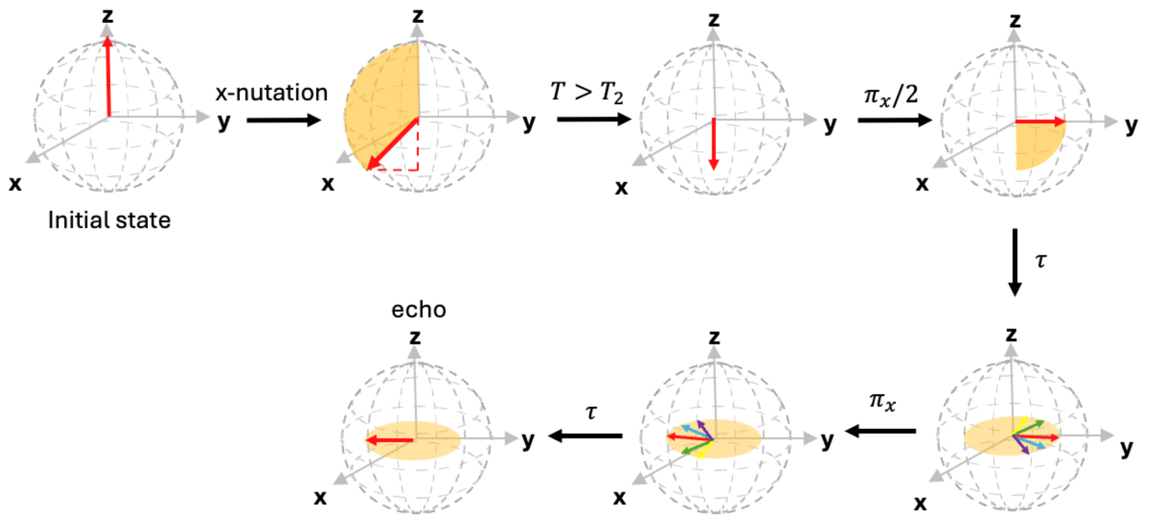

Figure S13. The evolution of electron spin polarization during the echo detected nutation experiment. The state is prepared with the x-nutation and spins rotates around z-axis. After time T > T_2 the coherence is lost, leaving only the net polarization along z, there the Hahn-echo sequence is employed to capture the z-projection caused by the x-nutation. The x-nutation pulse length is varied to produce the Rabi oscillation.

Carr-Purcell-Meiboom-Gill (CPMG) nuclear spin sensing: the measurements were performed with ($\pi_x/2$ – $\tau/2$ – [π_y -τ - π_y]^N - τ – echo) sequence. The basis set up follows the Hahn echo experiments. The basic pulse sequence of CPMG is shown in **Figure S14**.

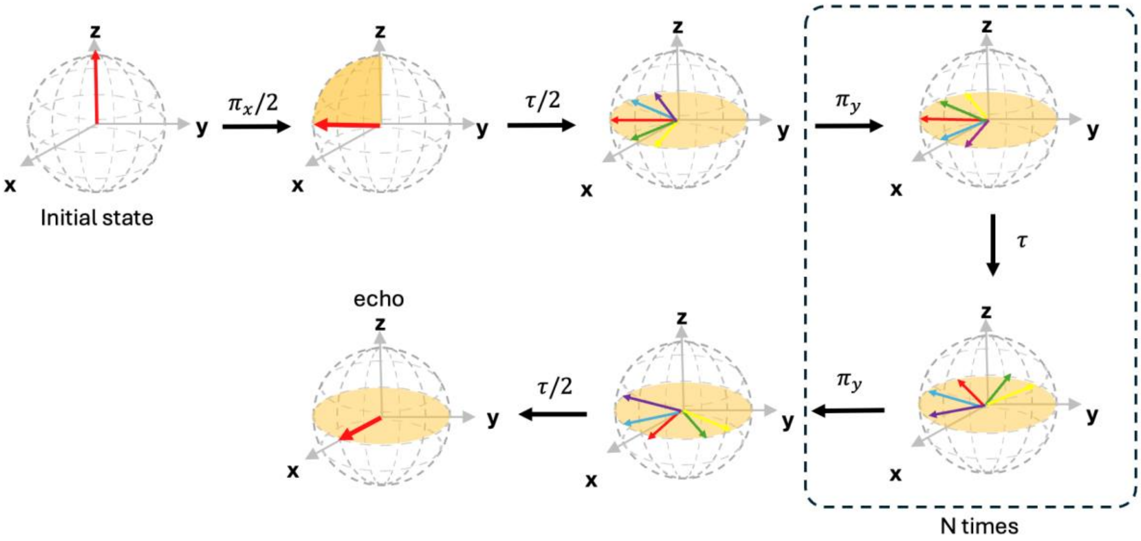

Figure S14. The evolution of spin under the CPGM nuclear spin sensing experiment. After the $\pi_x/2$, spins evolve freely in x-y plane with time delay $\tau/2$, then the π_y pulse flips the spin packet by 180 degrees around the y-axis. At the time delay τ, the spin further evolves in the x-y plane and then flips by another 180 degrees around the y-axis. This $\pi_y/2$- τ- $\pi_y/2$ sequence will be performed N times. The spin echo signal can be detected after time $\tau/2$. If τ is not in resonance with nuclear spin rotation, the nuclear spins are decoupled from the electron spin, resulting in a prolonged T_2. Alternatively, if τ is equal to a multiply of nuclear resonant time τ_I =1/($2\vartheta_x^{m_s}$) ($\vartheta_x^{m_s}$is the nuclear frequency), electron spin is coupled to the target nuclear spins, causing the dynamic decoupling to fall. This generates the decoherent dips in CPMG spectra, which can be used for spin sensing, or as the basis of dynamic nuclear spin register.
